# Different indicators of socioeconomic status and their relative importance as determinants of health in old age

**DOI:** 10.1186/s12939-017-0670-3

**Published:** 2017-09-26

**Authors:** Alexander Darin-Mattsson, Stefan Fors, Ingemar Kåreholt

**Affiliations:** 1 Aging Research Centre (ARC), Karolinska Institutet & Stockholm University, Gävlegatan 16, 113 30 Stockholm, Sweden; 2 Institute of Gerontology, School of Health and Welfare, Aging Research Network-Jönköping (ARN-J), Jönköping University, Jönköping, Sweden

**Keywords:** Socioeconomic indicators, Education, Social class, Income, Occupational complexity, SES-index, Late-life health

## Abstract

**Background:**

Socioeconomic status has been operationalised in a variety of ways, most commonly as education, social class, or income. In this study, we also use occupational complexity and a SES-index as alternative measures of socioeconomic status. Studies show that in analyses of health inequalities in the general population, the choice of indicators influence the magnitude of the observed inequalities. Less is known about the influence of indicator choice in studies of older adults. The aim of this study is twofold: i) to analyse the impact of the choice of socioeconomic status indicator on the observed health inequalities among older adults, ii) to explore whether different indicators of socioeconomic status are independently associated with health in old age.

**Methods:**

We combined data from two nationally representative Swedish surveys, providing more than 20 years of follow-up. Average marginal effects were estimated to compare the association between the five indicators of SES, and three late-life health outcomes: mobility limitations, limitations in activities of daily living (ADL), and psychological distress.

**Results:**

All socioeconomic status indicators were associated with late-life health; there were only minor differences in the effect sizes. Income was most strongly associated to all indicators of late-life health, the associations remained statistically significant when adjusting for the other indicators. In the fully adjusted models, education contributed to the model fits with 0–3% (depending on the outcome), social class with 0–1%, occupational complexity with 1–8%, and income with 3–18%.

**Conclusions:**

Our results indicate overlapping properties between socioeconomic status indicators in relation to late-life health. However, income is associated to late-life health independently of all other variables. Moreover, income did not perform substantially worse than the composite SES-index in capturing health variation. Thus, if the primary objective of including an indicator of socioeconomic status is to adjust the model for socioeconomic differences in late-life health rather than to analyse these inequalities per se, income may be the preferable indicator. If, on the other hand, the primary objective of a study is to analyse specific aspects of health inequalities, or the mechanisms that drive health inequalities, then the choice of indicator should be theoretically guided.

**Electronic supplementary material:**

The online version of this article (10.1186/s12939-017-0670-3) contains supplementary material, which is available to authorized users.

## Background

Studies of social determinants of health have repeatedly found socioeconomic inequalities in health. People with lower socioeconomic status (SES) have, on average, poorer health and die younger than those with more favourable SES. Socioeconomic inequalities in health persist into old age [1–4]. The results from a comparative study showed that the magnitude of inequality in morbidity in eleven European countries varied, however, health inequalities in all age groups were observed in all countries [3]. As most morbidity and mortality occurs in old age, these inequalities may affect a substantial proportion of the older population and increase the economic burden of public spending as the population ages.

In studies of health inequalities in later life, SES is most commonly operationalised as either education, social class, or income – and often without providing a rationale for the choice of indicator [5–7].

The overarching aim of this study was to explore how the three most common indicators of SES (education, social class, and income) are associated with health in old age. We also included occupational complexity as an alternative indicator of SES, as recent research suggests that complexity is a key driver of labour market stratification [8–10]. Education, social class, occupational complexity, and income all have overlapping properties, but they may also be independently associated with health in old age. Therefore, we explored the relation between these variables, a composite measure of the variables, and change in mobility limitations, activities of daily living, and psychological distress from working ages to old age.

### Education, social class, occupational complexity, income, and health in old age

The individuals’ highest attained level of education is generally reached in early adulthood, and serves to bridge socioeconomic conditions across generations [11]. Research suggest that the associations between education and health is driven by increases in human capital, psychosocial resources, living conditions, better health care and lifestyle, and selection (direct and indirect) [12]. Studies show that people with lower levels of education tend to have a more rapid health decline in old age [13]. However, educational level often show a weaker association with health in old age than other indices, such as wealth, income, tenure, and deprivation [14–16].

Most often, social class is identified using occupation as the stratifying principle. Many class schemas primarily distinguish occupations depending on ownership (i.e, between employers and employees). Thereafter, groups of employers and employees are distinguished depending on size and type of organization, skill requirements, power relations, and working conditions [17, 18]. Besides being associated with current income, social class is also associated with: i) income security, ii) short term income stability, and iii) long term income development [19, 20]. Results on the association between social class and late-life health are ambiguous. Duncan et al. [15] did not find any association between social class and mortality in older age, while other studies have [1, 21].

Some research have suggested that skill requirements, productivity and efficiency may provide a better explanation of social stratification at the labour market than conventional class theories [8–10]. Using Swedish data, Tåhlin found that the occupational complexity level is more strongly associated to the earned wages than traditional social class schemas [9, 10]. Tåhlin further argues that the level of occupational complexity associated with a given occupation can be used as a proxy for efficiency and productivity. Against this background, we have included a measure of occupational complexity as an alternative indicator of SES.

Income is often considered to be a straightforward indicator of material resources, and income is, robustly and positively, associated with longevity [22, 23]. Income might affect the health of people by enabling those with high income to lead healthy lifestyles, while those at the lower rungs of the income distribution have fewer of these enabling resources [24]. Income has an important role as an enabling resource for health care access and use in the Andersen health behavioural model [25, 26]. On the other hand, poor health can also result in lower incomes [27]. Hence, the association between income and health is likely shaped by bi-directional causal mechanisms. In the literature, measures indicating financial situation is commonly most strongly associated to health and mortality in old age, when compared to e.g. educational level and social class [14–16].

The rationale for including specific indicators of SES in studies of health in old age may vary. It could be to monitor, or to understand, how the social patterning of resources affect health. In this case, it is important to consider the different pathways and mechanism that education, social class, income, and occupational complexity might have on health. Another reason could be to merely adjust a model for as much socioeconomic variance as possible, without any specific interest in the mechanisms driving the health inequalities. Research suggest that a composite measures of individual level indicators could be appropriate for this purpose [28]. The findings from a previous study, based on the same data as the present study, suggested that a composite measure of individual level variables of SES was more suitable for this purpose than any of the individual variables [29]. On the other hand, using a composite measure of individual level variables of SES, may obscure the underlying mechanisms and prevent progression in the understanding of how different aspects of SES contributes to health.

Education, social class, occupational complexity, and income are inherently associated, as education is associated with occupational position and complexity, which in turn is associated with income [8, 30]. In addition, socioeconomic disadvantage tend to accumulate over the life course, both between and within socioeconomic domains [31]. Evidence suggest that there are certain, sensitive, periods in life where exposure to socioeconomic disadvantages may have increased and sustained effect on health throughout the life course [32].

Studies of the working age population have showed that different indicators of SES are differently associated with different health outcomes, which suggest that different underlying mechanisms may generate these associations [5, 33]. These findings suggest that the choice of indicator may be of importance for the results and the interpretations, when studying socioeconomic inequalities in health. Less is known of how different SES indicators relate to health in old age.

A thorough exploration of how the association between SES and health in old age varies by indicator of SES may provide important insights into the mechanisms generating socioeconomic inequalities in late-life health. As most commonly used indictors of SES are rooted in educational and occupational stratification, we chose to assess these variables in late working life when people tend to have reached their peak positions, in terms of educational level, social class, and income.

In the present study, late life health is assessed in terms of mobility limitations, limitations in activities of daily living (ADL), and psychological distress. These are all common areas of health problems in later life, and the burden of these afflictions in terms of societal costs is substantial [34]. Mobility limitations and ADL limitations are rare among people under the age of 40, but after the age of 40 the prevalence increase with age. Mobility and ADL limitations, and psychological distress are all strong predictors of need for social services and institutionalization [35–37]. Moreover, psychological distress also predict other health outcomes with special relevance for the old age population, such as dementia, mortality, cardiovascular diseases, and cerebrovascular disease [38–40].

## Methods

### Data

All analyses were conducted on linkages drawn from two data sources: the Swedish Level of Living Surveys (LNU) and the Swedish Longitudinal Study of Living Conditions of the Oldest Old (SWEOLD). Each linkage entail data from one wave of LNU with follow-up in a wave of SWEOLD. LNU and SWEOLD are both longitudinal social surveys, based on random samples of the Swedish population. The first wave of LNU was carried out in 1968 using a nationally representative sample of individuals aged 18 to 75 years. The sample was then followed-up in 1974, 1981, 1991, 2000, and 2010 [41]. The response rates for the waves used in the present study varies between 78.3% and 90.8% (n ≈ 6000–8000). In LNU, respondents were asked about a wide variety of topics, including their present health status, educational achievements, and occupation. SWEOLD is a continuation of LNU: it includes those who have ‘aged out’ of the LNU sample (i.e. those who are 75 years or older), and who were previously included in the LNU sample. SWEOLD has been conducted in 1992, 2002, 2004, and 2011; and the response rates varied between 84.4% and 95.4% (n ≈ 600–1000). In the 2004 wave, the age-span included was wider, and included those aged 69 years and older [42].

The independent variables were assessed at baseline and dependent variables in the designated follow-up. Respondents from LNU 1968 were followed-up in SWEOLD 1992 (linkage 1), respondents from LNU 1981 were followed-up in SWEOLD 2002 (linkage 2) and SWEOLD 2004 (linkage 3), and respondents from LNU 1991 were followed-up in SWEOLD 2011 (linkage 4).

The four linkages were analysed separately and as pooled data. The magnitude of the associations between the independent variables and the outcomes varied by linkage, but the differences were small and not systematic. Thus, we present the results from the full sample, with all the linkages pooled.

The study was restricted to people who were 46 to 64 years at baseline, and who were alive and participated in the designated follow-up (*n* = 2342). The age restriction was based on the possibility to be included in a follow-up in old age (SWEOLD data). Social class and occupational complexity level is based on occupation, therefore we included only those that were in paid employment (or self-employed) at baseline in the main analyses (*n* = 2027; 87% of the total sample). However, we performed separate analyses for those who were not in paid employment (or self-employed) at baseline (Table 3).

In the analyses of mobility limitations, the study sample was restricted to people without any mobility limitations at baseline (*n* = 1772), and in the analyses of psychological distress, to people without psychological distress at baseline (*n* = 1616). Sensitivity analyses, comparing the estimates to estimates based on the full sample showed similar results. Respondents who did not answer the questions about the outcome variables were excluded, which resulted in a different number of observations in the analyses of mobility limitations (*n* = 1763) and psychological distress (*n* = 1596). Since ADL limitations was not assessed in the LNU (baseline) all respondents were analysed studying ADL limitations (*n* = 2027).

### Measurements

*Mobility limitations* were assessed by the question: ‘Can you walk 100 meters at a fairly brisk pace without problems?’ and ‘Can you climb stairs (up and down) without problems?’ The response alternatives were ‘yes’ and ‘no’. Responses were summarised in an index that ranged from 0 (no problems) to 2 (problems with both tasks).

*Limitations in activity of daily living (ADL)* were assessed by five questions: ‘Can you eat by yourself?’, ‘Can you go to the toilet by yourself?’, ‘Can you dress and undress yourself?’, ‘Can you get in and out of bed by yourself?’, and ‘Can you wash your hair by yourself?’. The response alternatives were ‘yes, manage completely by myself’, ‘yes, with help’, and ‘no, not at all’. The responses were summarised in an index from 0 (managed all five tasks without help) to 10 (not able to perform any of the tasks).

*Psychological distress* was assessed by a general question: ‘Have you had any of the following diseases or disorders during the last 12 months?’, followed by a multi-item list of symptoms and disorders. We calculated an index based on the responses regarding anxiety and depressive symptoms. The response alternatives were ‘no’, ‘yes, slight’, and ‘yes, severe’. The responses were summarised in an index ranging from 0 (no problems) to 4 (severe psychological distress).

*Education* was measured as educational attainment. Self-reported educational attainment was divided into three groups: high, medium, or low. Upper secondary school or above was considered a high level of educational attainment. Compulsory school complemented with vocational training was considered a medium level of education. Attending compulsory school only or no schooling was considered as low level of education.

*Social class* was based on self-reported occupation at baseline, classified in accordance with the Swedish socioeconomic index (SEI), which is very similar to the Erikson, Goldthorpe, and Portocarero’s (EGP) schema [43]. The social classes were collapsed into three groups: low (unskilled and skilled blue-collar workers, small farmers and entrepreneurs without employees); medium (lower-level white-collar workers, farmers and entrepreneurs with 1 to 19 employees); and high (intermediate and upper-level white-collar workers, farmers and entrepreneurs with 20 or more employees, and academic professionals).

*Occupational complexity* was assessed by assigning a ‘substantive complexity’ score to each occupational category. The substantive complexity scores indicate the level of intellectual flexibility, engagement, and skills needed to perform working tasks of greater or lesser complexity. The measure of substantive complexity used in this study was developed by Roos and Treiman [44], and is based on the U.S. Dictionary of Occupational Titles (DOT) and the U.S. Census 1970. The DOT included 46 worker characteristics, assessed by job analysts. Roos and Treiman performed a principal component factor analysis and found a factor, including eight of the characteristics (general educational development, specific vocational preparation, complexity of work with data, intelligence aptitude, verbal aptitude, numerical aptitude, abstract interest in the job, and temperament for repetitive and continuous processes), that they called ‘substantive complexity’. This measure forms the basis for our measure of occupational complexity [44]. These scores have later been matched to Swedish occupational categories. See Andel et al. [45] for a description of the matching procedure and Darin-Mattsson et al. [29] for a more thorough description of occupational complexity. Occupational complexity ranges 0–10, the scale was divided into three categories on the complexity scale: 0–3.3 = low complexity, 3.4–6.4 = medium complexity, and 6.5–10 = high complexity.

*Individual income* was assessed from Swedish tax registers the year before baseline. Income was standardised to the purchasing power of 1991, log transformed and divided into quintiles. To increase the comparability of the models and to increase model fit, we divided income into three categories. We collapsed the two lowest quintiles into category 1 and quintiles three and four into category 2. Category 3 consisted of the fifth quintile, which included those with the highest incomes. This categorization was data-driven and based on tests of spline lines, which showed that the used categorization gave the best fit of the data.

We also used all of the SES indicators described above to construct a composite measure of SES (the *SES-index*)*.* We used education, social class, occupational complexity, and income as classified above, and summarized them. The index was then divided into tertiles. This was done to investigate whether a composite measure could, statistically, capture as much, or more, of the variance in late-life health as the individual indicators.

*Covariates* in all analyses were age, sex, and linkage. Age and sex was assessed by self-reports during the interview. Age was measured by birth year and given continuous representation in all analyses. Sex was categorized as either woman or man.

### Statistical methods

The results are presented in terms of Average Marginal Effects (AME) multiplied by 100. AMEs are estimated as the average difference in probability of the given outcome across all observations with covariates at their observed values. Thus, the estimates can be interpreted as the differences in the probability of the outcome in percentage points. We use AMEs, rather than odds ratios or β-coefficients, as they are comparable across models, intuitively interpretable, and provide absolute measures of inequality [46]. The AMEs from ordered logistic regression should be interpreted as the difference in the probability of reporting an outcome one-step higher on the scale than the outcome in the reference group. The proportional odds assumption was tested with the *gologit2* command in Stata [47], and the assumption was accepted.

In addition, we used McKelvey & Zavoina’s pseudo-R2 to compare estimates of explained variance from different models using the same dataset [48]. To study how each of the main independent variables contributed to model fit, we calculated the change in pseudo-R2 obtained by adding each independent variables (education, social class, income, and occupational complexity) one at a time to a model including only the outcomes and adjustments for sex, age, and linkage. For each indicator of socioeconomic status, we also calculated the change in pseudo-R2 associated to the exclusion of that variable from the full model (model 2).

We also ran all analyses stratified by sex. We found only small, statistically non-significant, differences between women and men except in the association between social class and mobility limitations (see results).

Our study design allow individuals to be included in several linkages. Thus, 282 people were included in both linkage 2 and 3 (<15% of the sample) and the indicators of health in old age could be clustered on individual level. As this could lead to artificially low standard errors, we used cluster-correlated robust estimates of variance in the analyses [49].

We also used multiple imputation to impute missing data on mobility limitations at baseline (113 imputations were included). Sensitivity analyses showed none or small differences between the imputed and non-imputed data. There were no internal non-responses of psychological distress at baseline in the analytical sample. No imputations were made on the outcome variables.

Spearman’s correlations between the independent variables were moderate. The correlation between education and social class was 0.51; between education and income, 0.38; between education and occupational complexity, 0.29; between class and income, 0.39; between class and occupational complexity, 0.38; and between income and occupational complexity, 0.22. All correlations were statistically significant (*p* < 0.001). Correlations between the indicators differed between the linkages, and there was a general trend towards minor, statistically non-significant, increases in the correlations over time. Model 2 was tested for multicollinearity with the *VIF* command in Stata, and the result indicated no multicollinearity.

## Results

Descriptive statistics are presented in Table 1. There were more women than men among the respondents, and women reported more problems in all three outcomes. Most respondents (around 50%) had low education and social class, but medium occupational complexity. The mean age at follow-up was 79 years. There were no statistically significant differences in psychological distress by age group. However, limitations in mobility and ADL increased with age. In general, the respondents with the highest level of the SES indicators had the least problems while the lowest group had the worst health outcomes, with one exception – people with medium social class had significantly lower ADL limitations than people with high or low social class. The association between ADL limitations and occupational complexity was not significant, and there were no statistically significant differences in psychological distress by education or social class.

**Table 1:** Descriptive statistics - proportion with no problems in mobility limitations, ADL limitations, and psychological distress and average number of problems for those reporting any problems

		Mobility limitations (0–2)^1^	ADL limitations (0–10)		Psychological distress (0–4)		
	All (*n* = 2036)	Mean problems^2^	*n* ^3^	Mean problems	*n*	Mean problems	*n*
Total	%	0.59	1763	0.53	2027	0.38	1596
Sex		*p = 0.005* ^*4*^		*p = 0.325*		*p < 0.001*	
* Women*	41.7	0.69	1000	0.57	1185	0.46	867
* Men*	58.3	0.48	763	0.48	842	0.27	729
Age at follow-up		*p < 0.001*		*p < 0.001*		*p = 0.797*	
* 69–75*	19.7	0.34	360	0.13	399	0.36	346
* 76–82*	56.8	0.58	1018	0.47	1153	0.39	884
* 83–88*	23.5	0.85	385	1.02	475	0.37	366
Education		*p < 0.001*		*p = 0.011*		*p = 0.105*	
* High*	9.4	0.35	182	0.28	191	0.25	159
* Medium*	36.1	0.51	668	0.48	730	0.40	584
* Low*	54.6	0.69	913	0.61	1106	0.38	853
Social class		*p < 0.001*		*p = 0.017*		*p = 0.135*	
* High*	24.4	0.44	458	0.62	495	0.32	425
* Medium*	22.2	0.56	400	0.40	450	0.38	343
* Low*	53.4	0.67	904	0.59	1078	0.41	826
Occupational complexity		*p = 0.001*		*p = 0.144*		*p = 0.009*	
* High*	11.4	0.39	216	0.46	233	0.25	193
* Medium*	51.3	0.59	897	0.43	1038	0.34	823
* Low*	37.2	0.65	650	0.71	756	0.48	580
Income		*p < 0.001*		*p = 0.031*		*p < 0.001*	
* High*	20.0	0.36	354	0.32	405	0.21	324
* Medium*	40.0	0.60	705	0.52	810	0.41	646
* Low*	40.0	0.69	704	0.66	812	0.43	646
SEP-index		*p < 0.001*		*p = 0.002*		*p = 0.017*	
* High*	29.9	0.42	558	0.35	606	0.30	503
* Medium*	36.2	0.61	635	0.49	727	0.41	576
* Low*	34.0	0.72	569	0.75	690	0.42	515

^1^Range of the dependent variable

^2^The average number of reported problems

^3^The number of observations differ between dependent variables because of internal non-response

^4^Kruskal-Wallis equality-of-populations rank test

The associations between education, social class, occupational complexity, income, the SES-index, and health in later life were all analysed separately in model 1 (Table 2), adjusted only for age, sex, and linkage. In model 2 (Table 2), all the SES indicators were analysed simultaneously in relation to each health outcome. We also adjusted the associations between each individual SES indicator and each health outcome, by the other indicators of SES one-by-one. For these results, see Additional file 1: Table S1 and Additional file 2: Table S2.

**Table 2:** Average marginal effects (AMEs) times 100 of reporting more health problems than the reference group and model fit (R2 change)

	Mobility limitations (*n* = 1763)^1^		R2 change	ADL limitations (*n* = 2036)		R2 change	Psychological distress (*n* = 1596)		R2 Change
	AME (%)	*p* value		AME (%)	*p* value		AME (%)	*p value*	
Education									
Model 1									
*-High*	(Ref)	[0.001] ^2^	12%^3^	(Ref)	[0.236]	3%	(Ref)	[0.060]	15%
*-Medium*	**7.02**	0.039		1.43	0.229		**9.46**	0.022	
*-Low*	**12.16**	0.001		1.99	0.103		**9.42**	0.022	
Model 2									
*-High*	(Ref)	[0.398]	1%^4^	(Ref)	[0.921]	0%	(Ref)	[0.468]	3%
*-Medium*	2.53	0.251		0.42	0.933		4.93	0.305	
*-Low*	5.08	0.531		0.12	0.767		2.87	0.568	
Social class									
Model 1									
*-High*	(Ref)	[0.001]	12%	(Ref)	[0.021]	5%	(Ref)	[0.104]	12%
*-Medium*	5.33	0.052		1.04	0.221		4.52	0.145	
*-Low*	**9.10**	0.000		**2.01**	0.007		**5.79**	0.035	
Model 2									
*-High*	(Ref)	[0.680]	0%	(Ref)	[0.937]	1%	(Ref)	[0.949]	1%
*-Medium*	−0.12	0.971		0.54	0.141		−0.83	0.829	
*-Low*	1.96	0.559		1.39	0.583		0.01	0.997	
Occupational complexity									
Model 1									
*-High*	(Ref)	[0.015]	9%	(Ref)	[0.043]	4%	(Ref)	[0.012]	18%
*-Medium*	**8.49**	0.004		0.26	0.153		4.95	0.181	
*-Low*	**10.15**	0.011		1.73	0.806		**10.34**	0.011	
Model 2									
*-High*	(Ref)	[0.510]	2%	(Ref)	[0.128]	1%	(Ref)	[0.094]	8%
*-Medium*	4.23	0.281		−1.03	0.400		2.42	0.567	
*-Low*	4.79	0.250		0.15	0.914		7.23	0.128	
Income									
Model 1									
*-High*	(Ref)	[0.000]	13%	(Ref)	[0.007]	7%	(Ref)	[0.006]	27%
*-Medium*	**10.37**	0.000		**2.44**	0.002		**10.58**	0.004	
*-Low*	**13.05**	0.000		**2.30**	0.007		**9.93**	0.002	
Model 2									
*-High*	(Ref)	[0.019]	7%	(Ref)	[0.102]	3%	(Ref)	[0.072]	18%
*-Medium*	**7.37**	0.005		**1.88**	0.025		**8.70**	0.037	
*-Low*	**9.31**	0.014		1.81	0.058		**8.42**	0.023	
SEP-index									
Model 1									
*-High*	(Ref)	[0.000]	16%	(Ref)	[0.008]	6%	(Ref)	[0.011]	20%
*-Medium*	**8.53**	0.000		**6.18**	0.021		**28.64**	0.003	
*-Low*	**10.38**	0.000		**9.56**	0.002		**20.09**	0.044	

Results in bold: *p* < 0.05

Model 1: adjusted for age, sex, and linkage. Model 2: model 1 + all independent variables were analyzed simultaneously

^1^Outcome variables have different scales: mobility limitations (0–2), ADL (0–10) and psychological distress (0–4)

^2^Numbers in square brackets [] are *p*-values for the contribution of the whole variable (likelihood ratio test)

^3^McKelvey & Zavoina’s pseudo-R2 change compared to a model without that specific indicator of SEP

^4^Pseudo-R2 change to model 2 attributed to that specific indicator of SEP

At this point, it is worth noting that the effect sizes in the main analyses are dependent on the scales of the outcome variables. As the health outcomes are scaled differently, this means that the estimates for the different outcomes are not comparable.

### Education

Education was significantly and negatively associated with both mobility limitations and psychological distress, but not with ADL limitations (Table 2; model 1). Including education increased the model fit by 12% in the analyses of mobility limitations, 3% for ADL limitations, and 15% for psychological distress. However, the inclusion of the variable did not significantly improve the model fit for ADL limitations and psychological distress. Only the high-educated group deviated significantly from the other groups, with better health. Adjusted for the other SES indicators (Table 2; model 2), education was no longer significantly associated with the health outcomes, and contributed with only 1% to the total model fit for mobility limitations, 0% for ADL limitations, and 3% for psychological distress (Table 2; model 2).

### Social class

People with low social class had an increased risk of all outcomes compared to the other two groups (Table 2; model 1). Social class contributed to the model fit with approximately the same magnitude as education. Analyses stratified by sex showed no differences in the probability of mobility limitations between men from the middle and upper social classes, however, the difference between women from the middle and upper social classes were statistically significant (AME 4.83, *p* = 0.002). In the fully adjusted model (Table 2; model 2), social class did not contribute to any explained variance at all.

### Occupational complexity

People who held occupations with a low complexity level had an elevated risk for mobility limitations and psychological distress in old age, compared to those who held an occupation characterised by high complexity. On the other hand, there were no statistically significant differences in ADL limitations, by complexity level. However, the inclusion of the variable improved the model fit significantly. Occupational complexity contributed less to the explained variance in the models than education, with the exception of the models for psychological distress. In the fully adjusted model (Table 2; model 2), there were no statistically significant associations between occupational complexity and late-life health.

### Income

Income was statistically significantly associated with all outcomes. The high-income group consequently had the lowest probabilities of adverse health of all income groups. Income contributed to model fit by 13% for mobility limitations, 7% for ADL limitations, and 27% for psychological distress (Table 2). Income was the only indicator that remained statistically significantly associated with the health outcomes in the fully adjusted models (model 2). In addition, income contributed the most to the model fits in model 2: 7% to mobility limitations, 3% to ADL limitations, and 18% to psychological distress.

### SES-index

The high SES-index group had better health than the other groups. The SES-index increased model fit more than any of the other indicators in the models of mobility limitations, about the same as income for ADL limitations, and less than income for psychological distress.

Including all the indicators of SES simultaneously increased model fit for mobility limitations by 29%, compared to a model only adjusted for age, sex, and linkage. The individual contribution of each variable sum up to an increased model fit of 46% (education 12% + social class 12% + occupational complexity 9% + income 13%). The difference between 46 and 29% indicate that the properties of the socioeconomic indicators overlap. The composite measure of the variables (SES-index) explained less variance than simultaneously including all SES indicators. A similar pattern emerged for ADL limitations. The contribution, when including all SES indicators simultaneously was 12%, while the sum of the individual contributions amounted to 19%, and the SES-index only explained 6%. The same pattern holds when psychological distress is the outcome, the summed increase in model fit for all SES indicators was 72%, whereas the combined increase was 37%, and the SES-index increased model fit by 20%. These result suggests that properties of the indicators overlap when analysing psychological distress.

### People not in paid employment at baseline

Only people who held a paid occupation at baseline were included in the above analyses (Table 2). In Table 3, we analysed those who did not have a paid occupation in relation to those who held a paid occupation at baseline.

**Table 3:** Average marginal effects (AME’s) multiplied by 100 for mobility limitations, ADL limitations, and psychological distress by paid occupation (including farmers and self-employed) or not at baseline

	Mobility limitations (*n* = 2010)^1^		ADL limitations (*n* = 2312)		Psychological distress (*n* = 1805)	
	AME (%)	*p* value	AME (%)	*p* value	AME (%)	*p* value
Paid occupation	(Ref)		(Ref)		(Ref)	
Not paid occupation^2^	**7.46**	0.024	**8.61**	0.000	**7.33**	0.024

Results in bold: *p* < 0.05. All models were adjusted for age, sex, and linkage

^1^The number of observations differ between dependent variables because of internal non-response

^2^People without a paid occupation at baseline. Not included in the main analyses

The results show that individuals who did not hold a paid occupation at baseline had higher probabilities of health problems in old age, compared to those who held paid occupations at baseline. The increased probabilities of health problems among those without a paid occupation at baseline was of the magnitude of 7.46 percentage points for mobility limitations, 8.62 percentage points for ADL limitations, and 7.33 percentage points for psychological distress.

## Discussion

We found that education, social class, occupational complexity, income, and a composite measure of SES (the SES-index) all were significantly associated with late life health. Income was most strongly associated with adverse health in late life. Income was also the only variable that remained statistically significantly associated with health when adjusted for the other separate SES variables. The results also indicate that income captured as much variance in the health outcomes as the composite measure, based on a series of SES indicators.

As with all studies, these results should be interpreted with caution. We explored the independent associations between several interrelated indicators of SES and late-life health in a mid-sized sample. Therefore, it is possible that the study lacked sufficient statistical power to efficiently detect all relevant associations.

As in all studies based on older adults, the results may be affected by selective survival [50]. As we studied inequality in health among those surviving into old age, inclusion in our target population was contingent on survival into old age. Thus, our estimates might be attenuated by the higher mortality rates among those with lower SES than among those with higher SES. Selective survival also implies that the indicators that exhibit the weakest associations with late-life health might be more strongly associated with health and mortality earlier in life, and vice versa. Similarly, there was also a selection into employment in mid-life that may affect our findings. This selection is typically stronger among women; as women are less likely than men to be employed in paid occupation. Hence, women were more likely than men to be excluded from our main analyses and end up in the not employed category. Our results show that those who were not employed at baseline were at an increased risk for poorer late-life health compared to those who were employed at baseline.

The study is based on data from social surveys. The health measures used are based on self-reports, rather than clinical examinations. It is not certain that the findings from this study can be generalized to other health outcomes. For example, the patterns might differ substantially for lifetime prevalence of coronary heart disease, cancer or diabetes.

We only explored individual-level variables of SES in this study. Previous research has suggested that, under some circumstances, household SES may be a more suitable indicator of women’s SES than individual SES [51]. As a sensitivity measure, we therefore also ran the models using household social class. The results were similar (all association went in the same direction and were approximately of the same magnitude), possibly because our main analyses were restricted to people with a paid occupation at baseline. We also tested household income for those cases where it was available. The associations between household income and individual income in relation to late-life health were similar.

Unfortunately, we were not able to assess wealth. Wealth is an indicator of financial resources accumulated over the life course (including inheritances), and the patterning of wealth in old age might therefore differ substantially from the patterning of incomes.

In 1939, Paul Lazarsfeld found that indices constructed with different information (that is, indices based on whether a person paid income tax, owned a car, or whether a person had a telephone in his or her home) resulted in approximately the same distribution of economic status throughout the population. He concluded that it is both theoretically and empirically probable that indices of economic status are interchangeable [52]. Geyer et al. [5] tested Lazarsfeld’s hypothesis using education, social class, and income as predictors of four different health outcomes in two populations aged 25–64 years. They found that each indicator had an independent effect on the outcomes but that the effect sizes and strengths of the associations varied by indicator. Education was the strongest predictor for diabetes and income strongest predicted all-cause mortality, while results were mixed for myocardial infarction morbidity and mortality. Thus, they argued that indicators of SES are not interchangeable in relation to health. In contrast, our results showed no independent effects by education, social class or occupational complexity on health in old age. Only income was independently associated with health in old age. In addition, Geyer and colleagues found that different indicators were differently associated to different outcomes. In contrast, our results showed that income was most strongly associated to all health outcomes, except compared with the SES-index. Torssander and Erikson [33] found similar results as Geyer and colleagues in relation to mortality risk in the Swedish population aged 35–59. Each indicator of SES was clearly associated with risk of death for both women and men. They suggested that each indicator’s crude association with mortality was in line with Lazarsfeld’s hypothesis on the interchangeability of indices, only if one assume that these indicators measure a general latent construct. On the other hand, the authors argue that if each indicator has an independent function, using them to map a latent construct would result in a loss of information [33]. This idea, that using one of these indicators interchangeably to indicate a latent concept of SES may result in a loss of information relevant for social stratification and health and policy implications, have been supported by others [6, 53]. Few studies have explored this issue in relation to health in old age, but Avlund et al. [14] found that different indicators of SES were individually associated to different health outcomes in old age. Our results show that income was the only indicator independently associated to late-life health, and that the indicators are otherwise statistically interchangeable. The indicators had approximately the same association to the outcomes, and their contribution to the model fits were comparable.

In line with previous studies [14–16], we also found that the most direct measure of economic differences (in this case, income) was most strongly and robustly associated with adverse health in old age.

A novel contribution of our study was the introduction of occupational complexity as an alternative indicator of SES in studies of health inequalities in old age. However, our results did not suggest that occupational complexity was a stronger determinant of late-life health than education, social class, income or a composite measure of SES (the SES-index).

Further research is needed to confirm the robustness of these findings and to explore the causal mechanisms underlying the associations between different aspects of socioeconomic status and late-life health.

## Conclusion

This study investigated the most commonly used indicators of SES in health research in relation to three health outcomes in old age. We also included a less traditional measure strongly associated with SES (occupational complexity), and a composite measure based on several indicators of SES (the SES-index). The study contributes to the literature by doing an in depth investigation of how the SES indicators relate to each other, and to late-life health, and by testing the predictive value of two novel measures of SES. In sum, our results suggests that income explain more variance in late-life health than any of the other SES indicators, with a predictive capacity that is equal, or even better, than that of a composite measure including a range of indicators. In sum, our results suggests that if the primary objective of including an indicator of SES, in studies of health in old age, is to merely adjust the model for socioeconomic differences income may be the preferred choice. If, on the other hand, the primary objective of the study is to examine health inequalities or the mechanisms that drive health inequalities in old age per se, then the choice of indicator should be made on the basis of a theoretical model that considers the unique properties of the different indicators.

## Additional files

Additional file 1: Table S1. Average marginal effects (AMEs) times 100 of reporting more health problems than the reference group and model fit (R2 change). (DOCX 17 kb)

Additional file 2: Table S2. Average marginal effects (AMEs) times 100 of reporting more health problems than the reference group and model fit (R2 change). (DOCX 17 kb)
