# Supplementary material for: Different indicators of socioeconomic status and their relative importance as determinants of health in old age
Source: Int J Equity Health. 2017 Sep 26;16:173. doi: 10.1186/s12939-017-0670-3 (PMC5615765; doi:10.1186/s12939-017-0670-3)
Supplement: Supplementary file 1 — Average marginal effects (AMEs) times 100 of reporting more health problems than the reference group and model fit (R2 change). (DOCX 17 kb) [file 12939_2017_670_MOESM1_ESM.docx]

| **Additional file 1: Table S1.** Average marginal effects (AMEs) times 100 of reporting more health problems than the reference group and model fit (R2 change). | | | | | | | | | | | | | | |
| --- | --- | --- | --- | --- | --- | --- | --- | --- | --- | --- | --- | --- | --- | --- |
| **Education** | **Model 1** | | R2 change^1^ | **Model 2** | | **Model 3** | | **Model 4** | | **Model 5** | | **Model 6** | | R2 change^2^ |
|  | AME (%) | *p* value |  | AME (%) | *p* value | AME (%) | *p* value | AME (%) | *p* value | AME (%) | *p* value | AME (%) | *p* value |  |
| **Mobility limitations (n=1763)** | | |  |  |  |  |  |  |  |  |  |  |  |  |
| *High* | (Ref) | [0.001]^3^ | 12% |  |  | (Ref) | [0.121] | (Ref) | [0.102] | (Ref) | [0.014] | (Ref) | [0.398] | 1% |
| *Medium* | **7.02** | 0.039 |  |  |  | 4.99 | 0.197 | 3.75 | 0.314 | 5.38 | 0.132 | 2.53 | 0.251 |  |
| *Low* | **12.16** | 0.001 |  |  |  | 8.27 | 0.055 | 7.25 | 0.066 | **9.86** | 0.010 | 5.08 | 0.531 |  |
| **ADL limitations (n=2036)** | |  |  |  |  |  |  |  |  |  |  |  |  |  |
| *High* | (Ref) | [0.236]^9^ | 3% |  |  | (Ref) | [0.808] | (Ref) | [0.397] | (Ref) | [0.057] | (Ref) | [0.921] | 0% |
| *Medium* | 1.43 | 0.229 |  |  |  | 0.90 | 0.515 | 1.26 | 0.182 | 0.70 | 0.593 | 0.42 | 0.933 |  |
| *Low* | 1.99 | 0.103 |  |  |  | 0.80 | 0.582 | 1.65 | 0.301 | 0.91 | 0.494 | 0.12 | 0.767 |  |
| **Psychological distress (n=1596)** | | |  |  |  |  |  |  |  |  |  |  |  |  |
| *High* | (Ref) | [0.060]^9^ | 15% |  |  | (Ref) | [0.197] | (Ref) | [0.401] | (Ref) | [0.120] | (Ref) | [0.468] | 3% |
| *Medium* | **9.46** | 0.022 |  |  |  | 8.12 | 0.076 | 6.21 | 0.289 | 7.62 | 0.073 | 4.93 | 0.305 |  |
| *Low* | **9.42** | 0.022 |  |  |  | 6.73 | 0.160 | 5.02 | 0.183 | 6.85 | 0.112 | 2.87 | 0.568 |  |
| **Social class** | | | | | | | | | | | | | | |
| **Mobility limitations (n=1763)** | | |  |  |  |  |  |  |  |  |  |  |  |  |
| *High* | (Ref) | [0.001] | 12% | (Ref) | [0.100] |  |  | (Ref) | [0.154] | (Ref) | [0.030] | (Ref) | [0.680] | 0% |
| *Medium* | 5.33 | 0.052 |  | 3.01 | 0.328 |  |  | 2.41 | 0.417 | 3.69 | 0.212 | -0.12 | 0.971 |  |
| *Low* | **9.10** | 0.000 |  | **6.04** | 0.038 |  |  | 5.20 | 0.062 | **7.15** | 0.010 | 1.96 | 0.559 |  |
| **ADL limitations (n=2036)** | |  |  |  |  |  |  |  |  |  |  |  |  |  |
| *High* | (Ref) | [0.021] | 5% | (Ref) | [0.091] |  |  | (Ref) | [0.028] | (Ref) | [0.211] | (Ref) | [0.937] | 1% |
| *Medium* | 1.04 | 0.221 |  | **0.88** | 0.038 |  |  | **1.06** | 0.009 | 1.30 | 0.113 | 0.54 | 0.141 |  |
| *Low* | **2.01** | 0.007 |  | 1.85 | 0.349 |  |  | 1.98 | 0.214 | 0.48 | 0.609 | 1.39 | 0.583 |  |
| **Psychological distress (n=1596)** | | |  |  |  |  |  |  |  |  |  |  |  |  |
| *High* | (Ref) | [0.104] | 12% | (Ref) | [0.411] |  |  | (Ref) | [0.726] | (Ref) | [0.616] | (Ref) | [0.949] | 1% |
| *Medium* | 4.52 | 0.145 |  | 2.63 | 0.436 |  |  | 1.88 | 0.586 | 2.11 | 0.327 | -0.83 | 0.829 |  |
| *Low* | **5.79** | 0.035 |  | 4.20 | 0.185 |  |  | 2.47 | 0.423 | 3.10 | 0.543 | 0.01 | 0.997 |  |

Results in bold: *p*<0.05. Model 1: adjusted for age, sex, and linkage. Model 2: adjusted for age, sex, linkage, and education. Model 3: adjusted for age, sex, linkage, and social class. Model 4: adjusted for age, sex, linkage, and occupational complexity. Model 5: adjusted for age, sex, linkage, and income. Model 6: all independent variables are analysed simultaneously.

^1^McKelvey & Zavoina's pseudo-R2 change compared to a model without that specific measure of SEP. ^2^Pseudo-R2 change to the full model attributed to that specific measure of SEP.

^3^Numbers in square brackets [] are p-values for the contribution of the whole variable (likelihood ratio test).
