# Supplementary material for: Different indicators of socioeconomic status and their relative importance as determinants of health in old age
Source: Int J Equity Health. 2017 Sep 26;16:173. doi: 10.1186/s12939-017-0670-3 (PMC5615765; doi:10.1186/s12939-017-0670-3)
Supplement: Supplementary file 2 — Average marginal effects (AMEs) times 100 of reporting more health problems than the reference group and model fit (R2 change). (DOCX 17 kb) [file 12939_2017_670_MOESM2_ESM.docx]

| **Additional file 2: Table S2.** Average marginal effects (AMEs) times 100 of reporting more health problems than the reference group and model fit (R2 change). | | | | | | | | | | | | | | |
| --- | --- | --- | --- | --- | --- | --- | --- | --- | --- | --- | --- | --- | --- | --- |
| **Occupational complexity** | **Model 1** | | R2 change^1^ | **Model 2** | | **Model 3** | | **Model 4** | | **Model 5** | | **Model 6** | | R2 change^2^ |
|  | AME (%) | *p* value |  | AME (%) | *p* value | AME (%) | *p* value | AME (%) | *p* value | AME (%) | *p* value | AME (%) | *p* value |  |
| **Mobility limitations (n=1763)** | | |  |  |  |  |  |  |  |  |  |  |  |  |
| *High* | (Ref) | [0.015]^3^ | 9% |  | [0.180] |  | [0.367] |  |  |  | [0.187] |  | [0.510] | 2% |
| *Medium* | **8.49** | 0.004 |  | 6.17 | 0.084 | 4.97 | 0.192 |  |  | 5.78 | 0.109 | 4.23 | 0.281 |  |
| *Low* | **10.15** | 0.011 |  | 6.89 | 0.067 | 5.74 | 0.159 |  |  | 6.89 | 0.067 | 4.79 | 0.250 |  |
| **ADL limitations (n=2036)** | |  |  |  |  |  |  |  |  |  |  |  |  |  |
| *High* | (Ref) | [0.043] | 4% |  | [0.084] |  | [0.114] |  |  |  | [0.098] |  | [0.128] | 1% |
| *Medium* | 0.26 | 0.153 |  | -0.07 | 0.950 | -0.93 | 0.437 |  |  | -0.29 | 0.792 | -1.03 | 0.400 |  |
| *Low* | 1.73 | 0.806 |  | 1.29 | 0.308 | 0.29 | 0.833 |  |  | 1.00 | 0.423 | 0.15 | 0.914 |  |
| **Psychological distress (n=1596)** | | |  |  |  |  |  |  |  |  |  |  |  |  |
| *High* | (Ref) | [0.012] | 18% |  | [0.049] |  | [0.072] |  |  |  | [0.052] |  | [0.094] | 8% |
| *Medium* | 4.95 | 0.181 |  | 3.21 | 0.402 | 3.49 | 0.403 |  |  | 2.88 | 0.459 | 2.42 | 0.567 |  |
| *Low* | **10.34** | 0.011 |  | 8.22 | 0.052 | 8.34 | 0.078 |  |  | 7.76 | 0.066 | 7.23 | 0.128 |  |
| **Income** | | | | | | | | | | | | | | |
| **Mobility limitations (n=1763)** | | |  |  |  |  |  |  |  |  |  |  |  |  |
| *High* | (Ref) | [0.000] | 13% |  | [0.003] |  | [0.006] |  | [0.000] |  |  |  | [0.019] | 7% |
| *Medium* | **10.37** | 0.000 |  | **8.44** | 0.001 | **8.27** | 0.002 | **9.06** | 0.001 |  |  | **7.37** | 0.005 |  |
| *Low* | **13.05** | 0.000 |  | **10.50** | 0.003 | **10.39** | 0.005 | **11.06** | 0.000 |  |  | **9.31** | 0.014 |  |
| **ADL limitations (n=2036)** | |  |  |  |  |  |  |  |  |  |  |  |  |  |
| *High* | (Ref) | [0.007] | 7% | (Ref) | [0.019] | (Ref) | [0.074] | (Ref) | [0.012] |  |  | (Ref) | [0.102] | 3% |
| *Medium* | **2.44** | 0.002 |  | **2.26** | 0.005 | **1.96** | 0.022 | **2.26** | 0.010 |  |  | **1.88** | 0.025 |  |
| *Low* | **2.30** | 0.007 |  | **2.09** | 0.020 | 1.80 | 0.058 | **2.20** | 0.003 |  |  | 1.81 | 0.058 |  |
| **Psychological distress (n=1596)** | | |  |  |  |  |  |  |  |  |  |  |  |  |
| *High* | (Ref) | [0.006] | 27% | (Ref) | [0.040] | (Ref) | [0.039] | (Ref) | [0.023] |  |  | (Ref) | [0.072] | 18% |
| *Medium* | **10.58** | 0.004 |  | **9.24** | 0.012 | **9.55** | 0.025 | **9.28** | 0.007 |  |  | **8.70** | 0.037 |  |
| *Low* | **9.93** | 0.002 |  | **8.66** | 0.025 | **8.76** | 0.011 | **8.74** | 0.014 |  |  | **8.42** | 0.023 |  |

Results in bold: *p*<0.05. Model 1: adjusted for age, sex, and linkage. Model 2: adjusted for age, sex, linkage, and education. Model 3: adjusted for age, sex, linkage, and social class. Model 4: adjusted for age, sex, linkage, and occupational complexity. Model 5: adjusted for age, sex, linkage, and income. Model 6: all independent variables are analysed simultaneously.

^1^McKelvey & Zavoina's pseudo-R2 change compared to a model without that specific measure of SEP. ^2^Pseudo-R2 change to the full model attributed to that specific measure of SEP. ^3^Numbers in square brackets [] are p-values for the contribution of the whole variable (likelihood ratio test).
